# 10-epi-Protectin DX and Resolvin D5_n-3 DPA_ Attenuate Multi-Organ Inflammatory Injury in an LPS-Induced Murine Endotoxemia Model

**DOI:** 10.3390/ijms27083356

**Published:** 2026-04-08

**Authors:** Suyeon Kim, Uijin Kim, Nahyun Kim, Tae-Eui Lee, Jin Lee, Deok-Kun Oh, Ha Youn Shin

**Affiliations:** 1 Department of Biomedical Science and Engineering, Konkuk University, Seoul 05029, Republic of Korea; 0924tndus123@naver.com (S.K.); rladmlwls135@naver.com (U.K.); knh64@naver.com (N.K.); 2 School of Advanced Biotechnology, Konkuk University, Seoul 05029, Republic of Korea; 3 Terasaki Institute for Biomedical Innovation, Woodland Hills, CA 91367, USA; 4 Department of Bioscience and Biotechnology, Konkuk University, Seoul 05029, Republic of Korea; legals1@naver.com (T.-E.L.); gin3071@naver.com (J.L.); deokkun@konkuk.ac.kr (D.-K.O.)

**Keywords:** sepsis, endotoxemia, oxylipins, 10-epi-Protectin DX (10-epi-PDX), Resolvin D5_n-3 DPA_ (RvD5_n-3 DPA_), lipopolysaccharide (LPS), inflammation

## Abstract

Sepsis is a life-threatening syndrome driven by dysregulated immune activation and multi-organ dysfunction, with limited effective therapies. Oxylipins are endogenous lipid mediators that promote the resolution of inflammation and tissue repair, yet their therapeutic potential in systemic inflammatory diseases remains incompletely understood. In this study, we evaluated the effects of two oxylipins, 10-epi-Protectin DX (10-epi-PDX) and Resolvin D5_n-3 DPA_ (RvD5_n-3 DPA_), in a lipopolysaccharide (LPS)-induced murine endotoxemia model. Given that this model recapitulates key features of systemic inflammation and multi-organ injury relevant to sepsis-associated conditions, oxylipin effects were assessed across major organs implicated in systemic inflammatory pathology. Administration of either oxylipin significantly reduced systemic tissue injury and inflammatory damage in the lungs, kidneys, and liver. These protective effects were accompanied by suppression of inflammatory responses and marked improvements in histopathological outcomes. These findings indicate that 10-epi-PDX and RvD5_n-3 DPA_ possess organ-protective, anti-inflammatory properties in endotoxemia and support further investigation of their potential as therapeutic candidates for limiting systemic inflammatory injury.

## 1. Introduction

Sepsis is a life-threatening condition arising from a dysregulated host response to microbial infection, characterized by systemic inflammation and progressive organ dysfunction [1,2]. Despite advances in supportive care, sepsis remains a leading cause of mortality worldwide, accounting for approximately 20% of all global deaths and imposing a substantial public health burden, with tens of millions of cases occurring annually [1,3]. The pathophysiology of sepsis involves elevated cytokine production, vascular leakage, hypotension, and progression to multiple organ failure [1,2]. However, current clinical management remains largely limited to infection control and supportive measures aimed at preserving organ function, as no approved disease-specific therapies for sepsis are currently available [3,4]. Collectively, these observations highlight a critical unmet need for therapeutic strategies that can effectively modulate inflammation while promoting immune resolution.

In this context, oxylipins have emerged as important regulators of inflammatory responses. Oxylipins are endogenously derived bioactive lipid mediators generated from polyunsaturated fatty acids through distinct enzymatic pathways, producing structurally and functionally diverse molecules with specific biological activities [5,6]. These lipid mediators regulate a wide range of cellular processes, including innate and adaptive immune responses, and encompass several families such as protectins, resolvins, maresins, and lipoxins [7,8,9].

Protectin DX (PDX), a docosahexaenoic acid (DHA)-derived oxylipin belonging to the protectin family, has been shown to exert anti-inflammatory and tissue-protective effects, in part through suppression of pro-inflammatory cytokine production [10,11,12]. In contrast, the biological functions and therapeutic potential of its stereoisomer, 10-epi-Protectin DX (10-epi-PDX), remain largely unexplored [13]. In parallel, Resolvin D5_n-3 DPA_ (RvD5_n-3 DPA_), a resolvin-family oxylipin derived from n-3 docosapentaenoic acid, has been reported to exhibit anti-inflammatory, pro-resolving, and tissue-protective properties across multiple inflammatory settings [14,15,16,17]. However, its role in sepsis-associated inflammation and the molecular mechanisms underlying its actions have yet to be defined.

In the present study, we employed a well-established lipopolysaccharide (LPS)-induced mouse endotoxemia model, which recapitulates key pathophysiological features of systemic inflammation and multi-organ injury associated with sepsis, to investigate the therapeutic effects of 10-epi-PDX and RvD5_n-3 DPA_ [18,19]. Sepsis is a multi-organ disorder with prominent involvement of the lungs, kidneys, and liver, frequently progressing to acute respiratory distress syndrome, acute lung injury, acute kidney injury, and hepatic dysfunction [20]. Accordingly, we evaluated the immunomodulatory and tissue-protective actions of these oxylipins across multiple organs. Through this integrated approach, we aimed to define the therapeutic potential and mechanistic basis of 10-epi-PDX and RvD5_n-3 DPA_, thereby providing a rationale for inflammation-resolution-based therapeutic strategies in systemic inflammatory conditions relevant to sepsis.

## 2. Results

### 2.1. Protective Effects of 10-epi-PDX and RvD5n-3 DPA Against LPS-Induced Endotoxemia Pathology

**Figure 1:**
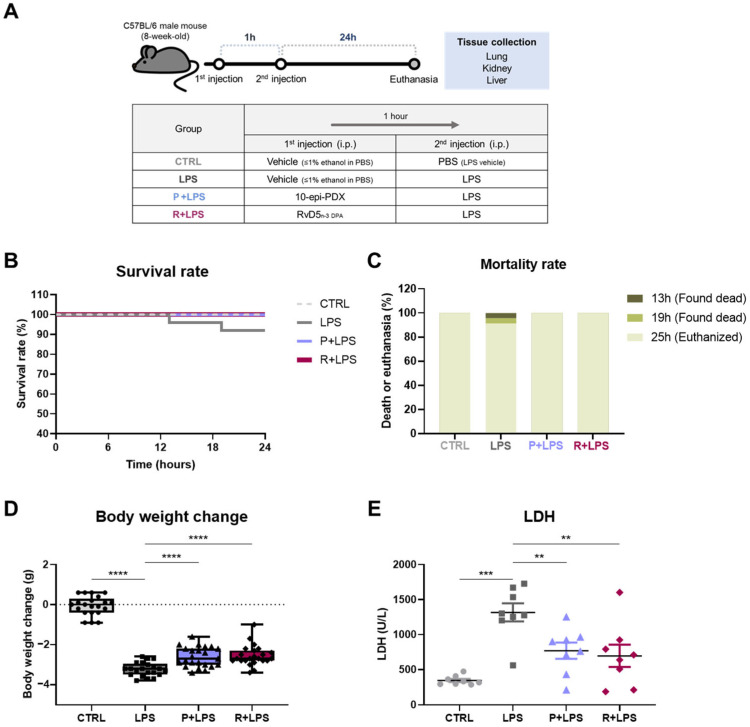
10-epi-PDX and RvD5_n-3 DPA_ mitigate LPS-induced mortality and body weight loss in mice. (**A**) Schematic of the experimental design used to evaluate the protective effects of 10-epi-PDX and RvD5_n-3 DPA_ in an LPS-induced endotoxemia model. Mice were administered oxylipins or vehicle intraperitoneally, followed 1 h later by intraperitoneal LPS injection. Mice were sacrificed 24 h after LPS administration. (**B**,**C**) Survival rates (**B**) and mortality rates (**C**) of mice in each group (CTRL, *n* = 21; LPS, *n* = 26; P+LPS, *n* = 23; R+LPS, *n* = 23). (**D**) Body weight changes in mice (CTRL, *n* = 21; LPS, *n* = 24; P+LPS, *n* = 23; R+LPS, *n* = 23). (**E**) Serum LDH levels (CTRL, *n* = 8; LPS, *n* = 8; P+LPS, *n* = 8; R+LPS, *n* = 8). Data are presented as mean ± SEM. Statistical significance was determined by one-way ANOVA followed by Sidak’s post hoc test. ** *p* < 0.01, *** *p* < 0.001, **** *p* < 0.0001.

To evaluate the protective effects of 10-epi-PDX and RvD5_n-3 DPA_, an LPS-induced murine model of systemic inflammation was established. Mice were intraperitoneally administered LPS in the presence or absence of 10-epi-PDX or RvD5_n-3 DPA_, and survival and body weight were monitored to assess overall physiological status (Figure 1A). LPS challenge resulted in a mortality rate of 8.7%, whereas no mortality was observed in the control (CTRL) group (Figure 1B,C). Notably, treatment with either 10-epi-PDX or RvD5_n-3 DPA_ completely prevented LPS-induced mortality, resulting in 100% survival. Consistent with systemic illness, LPS-treated mice exhibited a marked body weight loss of 13.6%. In contrast, administration of 10-epi-PDX or RvD5_n-3 DPA_ significantly attenuated weight loss, limiting reductions to 11.3% and 11.2%, respectively, compared with CTRL animals (Figure 1D).

Because elevated circulating LDH levels are widely used as a marker of tissue injury, including in sepsis [21,22], serum LDH levels were next assessed. LDH is a cytosolic enzyme that catalyzes the final step of glycolysis and is released into the circulation upon cellular damage. Serum LDH levels were significantly increased in LPS-challenged mice compared with CTRL animals (Figure 1E). Importantly, treatment with either 10-epi-PDX or RvD5_n-3 DPA_ significantly reduced serum LDH levels, indicating attenuation of LPS-induced tissue injury. Collectively, these results suggest that 10-epi-PDX and RvD5_n-3 DPA_ confer protection against LPS-induced pathology, as evidenced by improved survival, reduced body weight loss, and decreased systemic tissue damage.

### 2.2. Suppression of LPS-Induced Lung Injury and Inflammation by 10-epi-PDX and RvD5_n-3 DPA_

**Figure 2:**
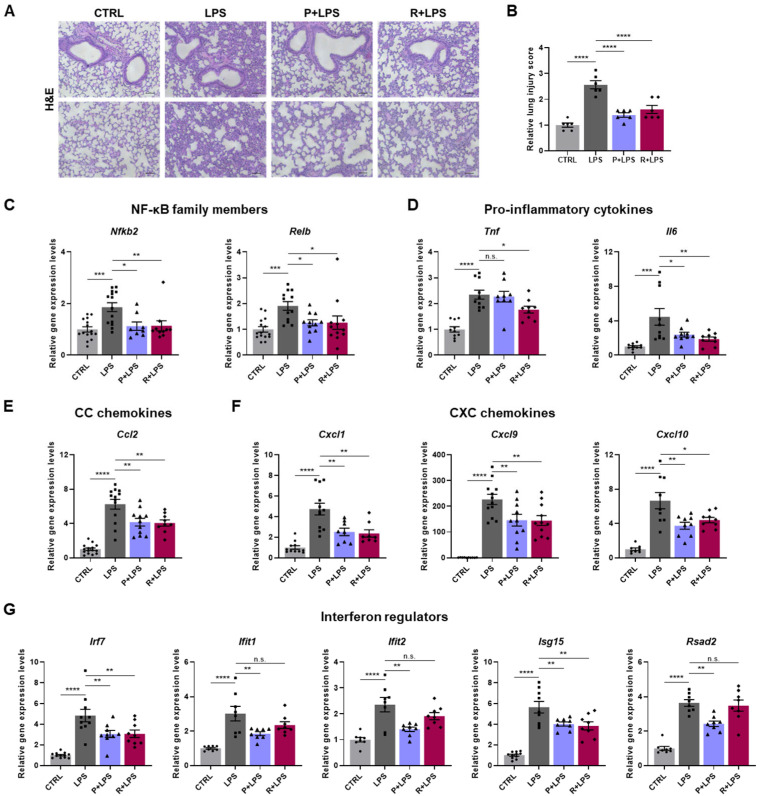
10-epi-PDX and RvD5_n-3 DPA_ alleviate LPS-induced lung inflammation. (**A**) Representative H&E-stained lung sections from each group (magnification, 100×; scale bars, 10 μm). (**B**) Quantification of lung injury scores (CTRL, *n* = 6; LPS, *n* = 6; P+LPS, *n* = 6; R+LPS, *n* = 6). (**C**–**G**) Relative mRNA expression levels of NF-κB subunit genes (**C**), pro-inflammatory cytokines (**D**), CC chemokines (**E**), CXC chemokines (**F**), and interferon-regulated genes (**G**) in lung tissue. Gene expression was normalized to *Gapdh* (CTRL, *n* ≥ 8; LPS, *n* ≥ 8; P+LPS, *n* ≥ 8; R+LPS, *n* ≥ 8). Data are presented as mean ± SEM. Statistical significance was determined by one-way ANOVA followed by Sidak’s post hoc test. * *p* < 0.05, ** *p* < 0.01, *** *p* < 0.001, **** *p* < 0.0001; n.s., not significant.

Sepsis is a systemic inflammatory disease caused by severe infection, in which the lungs are particularly vulnerable and frequently develop acute lung injury (ALI) and acute respiratory distress syndrome (ARDS) [23,24]. As lung injury is a key component of systemic inflammatory responses in sepsis, we examined the effects of 10-epi-PDX and RvD5_n-3 DPA_ on LPS-induced lung pathology. Histological analysis of hematoxylin and eosin (H&E)-stained lung sections revealed pronounced immune cell infiltration in the perialveolar regions of LPS-challenged mice, which was markedly attenuated by treatment with either 10-epi-PDX or RvD5_n-3 DPA_ (Figure 2A). Consistently, lung injury scores were significantly reduced in oxylipin-treated mice compared with the LPS group (Figure 2B).

**Figure 3:**
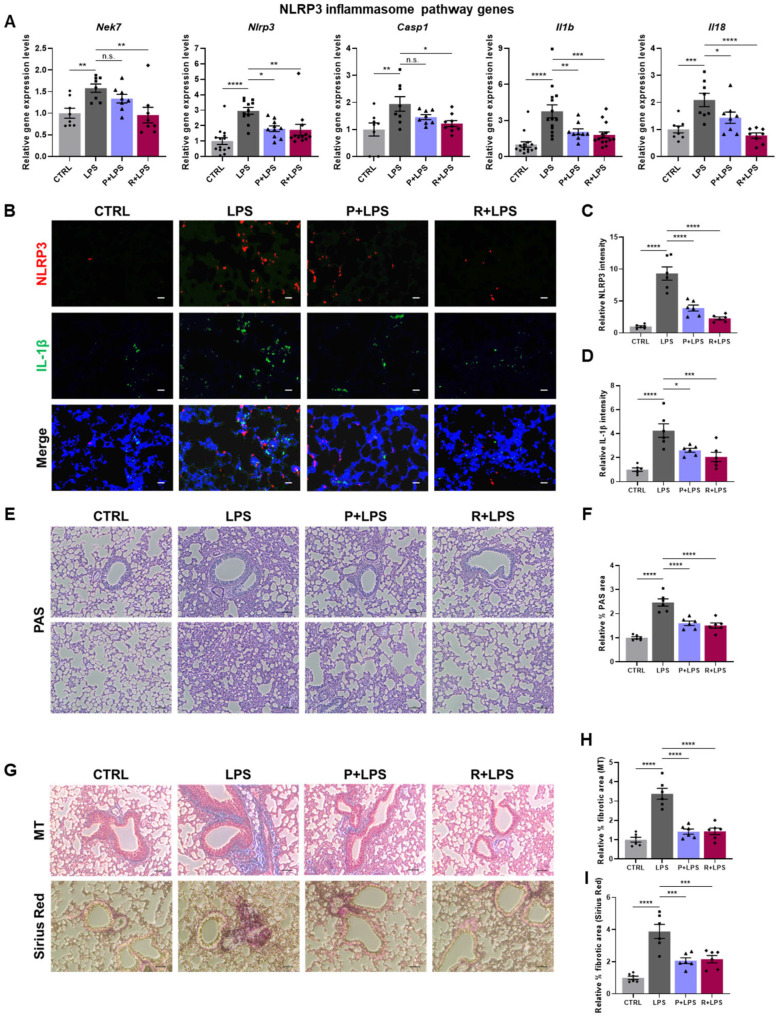
10-epi-PDX and RvD5_n-3 DPA_ suppress NLRP3 inflammasome activation, mucus accumulation, and fibrotic features in mouse lung tissue. (**A**) Relative mRNA expression levels of NLRP3 inflammasome-related genes in lung tissue (CTRL, *n* ≥ 8; LPS, *n* ≥ 8; P+LPS, *n* ≥ 8; R+LPS, *n* ≥ 8). (**B**) Representative immunofluorescence images showing protein expression of NLRP3 and IL-1β in lung tissue (magnification, 200×; scale bar, 100 μm). (**C**,**D**) Quantification of relative NLRP3 (**C**) and IL-1β (**D**) fluorescence intensity (CTRL, *n* = 6; LPS, *n* = 6; P+LPS, *n* = 6; R+LPS, *n* = 6). (**E**) Representative images of PAS-stained lung sections showing mucus accumulation (magnification, 100×; scale bar, 10 μm). (**F**) Quantification of PAS-positive areas in lung tissue (CTRL, *n* = 6; LPS, *n* = 6; P+LPS, *n* = 6; R+LPS, *n* = 6). (**G**) Representative images of MT-stained and Sirius Red-stained lung sections showing collagen deposition (magnification, 100×; scale bar, 10 μm). (**H**,**I**) Quantification of fibrotic areas in MT-stained (**H**) and Sirius Red-stained (**I**) lung sections (CTRL, *n* = 6; LPS, *n* = 6; P+LPS, *n* = 6; R+LPS, *n* = 6). Data are presented as mean ± SEM. Statistical significance was determined by one-way ANOVA followed by Sidak’s post hoc test. * *p* < 0.05, ** *p* < 0.01, *** *p* < 0.001, **** *p* < 0.0001; n.s., not significant.

To further assess inflammatory responses, we analyzed the expression of immune-related genes in lung tissue. LPS robustly induced the expression of NF-κB subunit genes (*Nfkb2* and *Relb*); pro-inflammatory cytokines (*Tnf* and *Il6*); chemokines, including CC chemokine (*Ccl2*) and CXC chemokines (*Cxcl1*, *Cxcl9*, and *Cxcl10*); and interferon-regulated genes (*Irf7*, *Ifit1*, *Ifit2*, *Isg15*, and *Rsad2*). Notably, the induction of these genes was markedly suppressed by oxylipin treatment (Figure 2C–G). Collectively, these findings indicate that 10-epi-PDX and RvD5_n-3 DPA_ attenuate LPS-induced pulmonary inflammatory responses. Given prior evidence that PDX and RvD5_n-3 DPA_ suppress NLRP3 inflammasome activation [17,25], we next examined the involvement of this pathway in LPS-induced lung pathology. LPS challenge significantly increased the expression of NLRP3 inflammasome-associated genes, including *Nek7*, *Nlrp3*, *Casp1*, *Il1b*, and *Il18*, in lung tissue (Figure 3A). This transcriptional activation was markedly suppressed by oxylipin treatment, with RvD5_n-3 DPA_ exerting the strongest inhibitory effect. Consistent with these transcriptional changes, immunofluorescence analysis revealed pronounced upregulation of NLRP3 and IL-1β protein levels in LPS-treated lungs, both of which were substantially reduced following administration of 10-epi-PDX or RvD5_n-3 DPA_ (Figure 3B–D).

### 2.3. Attenuation of LPS-Induced Pulmonary Mucus Accumulation and Fibrotic Features by 10-epi-PDX and RvD5_n-3 DPA_

Because excessive pulmonary inflammation is a known driver of mucus hypersecretion and fibrotic remodeling, including in sepsis, we next assessed mucus production in response to LPS challenge [26]. Periodic acid–Schiff (PAS) staining revealed a marked increase in mucus accumulation in the lungs of LPS-challenged mice, with more than a 2-fold expansion in PAS-positive areas compared with CTRL mice (Figure 3E,F). This aberrant mucus accumulation was significantly attenuated by oxylipin treatment.

We next evaluated fibrotic responses by assessing collagen deposition using Masson’s trichrome (MT) and Sirius Red staining. MT staining revealed extensive collagen accumulation in the lungs of LPS-treated mice, which was markedly reduced following treatment with either 10-epi-PDX or RvD5_n-3 DPA_ (Figure 3G and Figure S1A). Sirius Red staining yielded comparable results, confirming increased collagen deposition after LPS exposure and its significant attenuation by oxylipin treatment (Figure 3G and Figure S1B). Quantitative analyses corroborated these findings, demonstrating increased fibrotic areas in LPS-treated lungs relative to CTRLs and a pronounced reduction in oxylipin-treated groups (Figure 3H,I). Collectively, these results indicate that 10-epi-PDX and RvD5_n-3 DPA_ attenuate LPS-induced pulmonary mucus accumulation and fibrotic remodeling.

### 2.4. Reduced Renal Injury and Inflammation Following LPS Challenge by 10-epi-PDX and RvD5_n-3 DPA_

**Figure 4:**
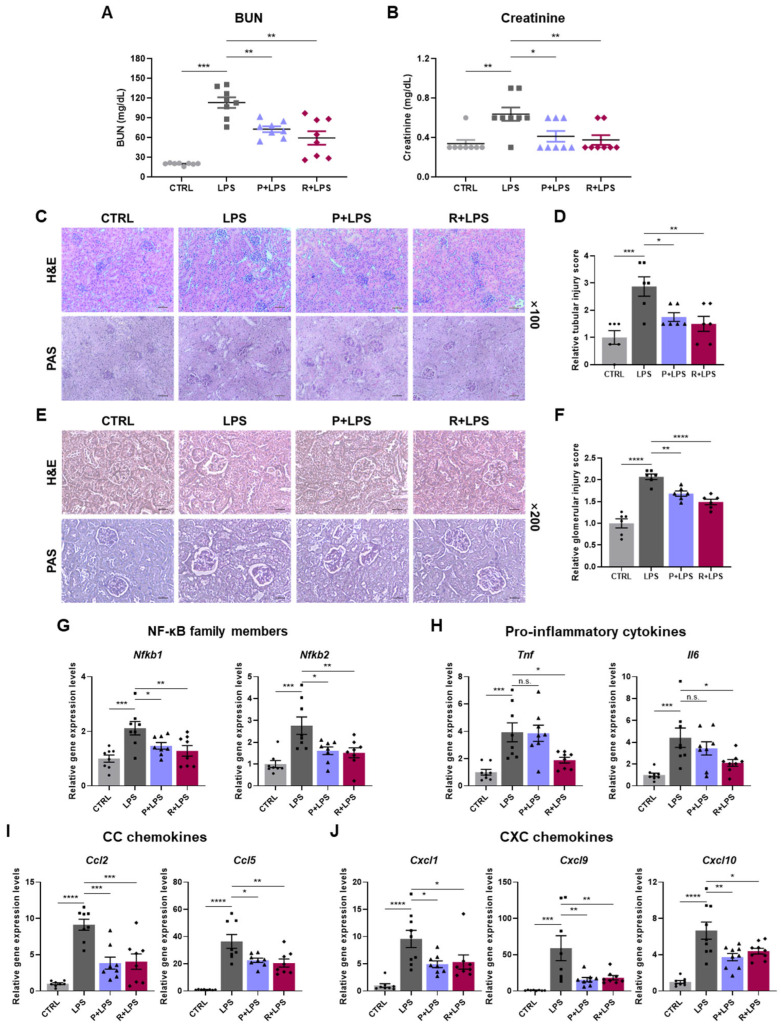
10-epi-PDX and RvD5_n-3 DPA_ ameliorate LPS-induced kidney injury and inflammation. (**A**,**B**) Serum BUN (**A**) and creatinine (**B**) levels in mice (CTRL, *n* = 8; LPS, *n* = 8; P+LPS, *n* = 8; R+LPS, *n* = 8). (**C**) Representative H&E- and PAS-stained kidney sections (magnification, 40×; scale bars, 10 μm). (**D**) Quantification of tubular injury scores (CTRL, *n* = 6; LPS, *n* = 6; P+LPS, *n* = 6; R+LPS, *n* = 6). (**E**) Representative H&E- and PAS-stained kidney sections (magnification, 100×; scale bar, 10 μm). (**F**) Quantification of glomerular injury scores (CTRL, *n* = 6; LPS, *n* = 6; P+LPS, *n* = 6; R+LPS, *n* = 6). (**G**–**J**) Relative mRNA expression levels of NF-κB subunit genes (**G**), pro-inflammatory cytokines (**H**), CC chemokines (**I**), and CXC chemokines (**J**) in kidney tissue (CTRL, *n* ≥ 8; LPS, *n* ≥ 8; P+LPS, *n* ≥ 8; R+LPS, *n* ≥ 8). Data are presented as mean ± SEM. Statistical significance was determined by one-way ANOVA followed by Sidak’s post hoc test. * *p* < 0.05, ** *p* < 0.01, *** *p* < 0.001, **** *p* < 0.0001; n.s., not significant.

Acute kidney injury (AKI) is a common complication of systemic inflammatory conditions, such as sepsis [27]. We therefore examined renal injury in the LPS-induced endotoxemia model. Consistent with AKI-associated dysfunction, LPS administration significantly increased serum blood urea nitrogen (BUN) and creatinine levels [28,29]. Treatment with either 10-epi-PDX or RvD5_n-3 DPA_ markedly attenuated these elevations, indicating protection against LPS-induced renal dysfunction (Figure 4A,B). Histological analyses further supported these findings. H&E and PAS staining revealed renal tubular damage and glomerular injury in LPS-treated mice, both of which were significantly ameliorated by oxylipin treatment (Figure 4C–F).

**Figure 5:**
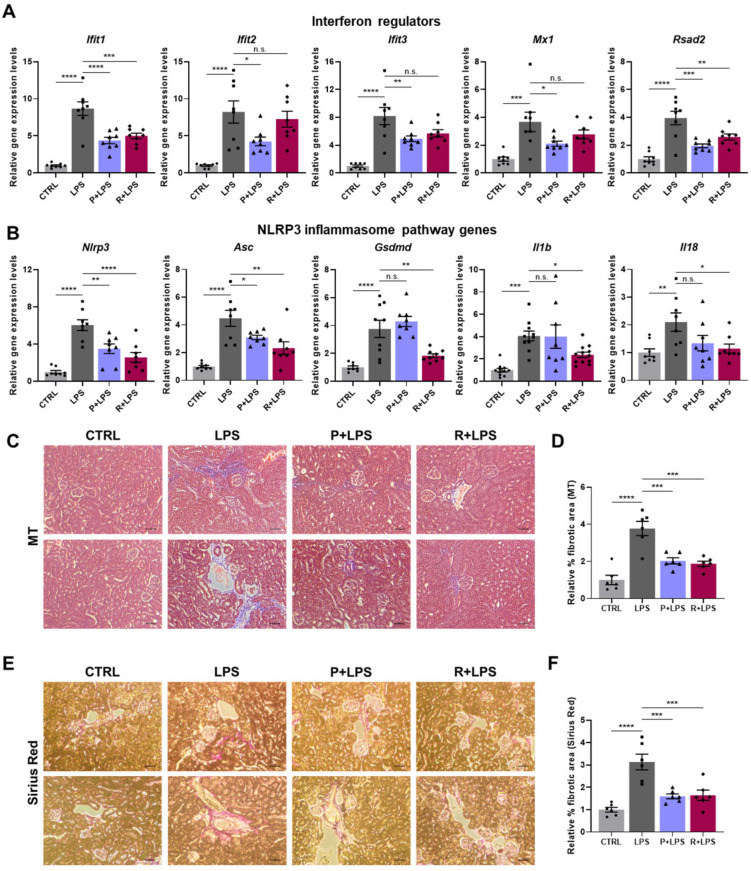
10-epi-PDX and RvD5_n-3 DPA_ attenuate NLRP3 inflammasome activation and ECM expansion in kidney tissue. (**A**,**B**) Relative mRNA expression levels of interferon regulatory genes (**A**) and NLRP3 inflammasome-related genes (**B**) in kidney tissue (CTRL, *n* ≥ 8; LPS, *n* ≥ 8; P+LPS, *n* ≥ 8; R+LPS, *n* ≥ 8). (**C**) Representative images of MT-stained kidney sections (magnification, 100×; scale bar, 10 μm). (**D**) Quantification of fibrotic areas in MT-stained sections (CTRL, *n* = 6; LPS, *n* = 6; P+LPS, *n* = 6; R+LPS, *n* = 6). (**E**) Representative images of Sirius Red-stained kidney sections (magnification, 100×; scale bar, 10 μm). (**F**) Quantification of fibrotic areas from Sirius Red-stained sections (CTRL, *n* = 6; LPS, *n* = 6; P+LPS, *n* = 6; R+LPS, *n* = 6). Data are presented as mean ± SEM. Statistical significance was determined by one-way ANOVA followed by Sidak’s post hoc test. * *p* < 0.05, ** *p* < 0.01, *** *p* < 0.001, **** *p* < 0.0001; n.s., not significant.

To characterize renal inflammatory responses, we next assessed the expression of immune-related genes in kidney tissue. LPS markedly upregulated NF-κB subunit genes (*Nfkb1* and *Nfkb2*), pro-inflammatory cytokines (*Tnf* and *Il6*), and multiple chemokines, including CC chemokines (*Ccl2* and *Ccl5*) and CXC chemokines (*Cxcl1*, *Cxcl9*, and *Cxcl10*) (Figure 4G–J). This inflammatory gene program was broadly suppressed by oxylipin treatment. In parallel, interferon-stimulated genes (*Ifit1*, *Ifit2*, *Ifit3*, *Mx1*, and *Rsad2*) were markedly induced following LPS challenge and were significantly reduced by oxylipin administration, with 10-epi-PDX exhibiting particularly strong suppressive effects (Figure 5A). Consistent with the lung findings, components of the NLRP3 inflammasome pathway (*Nlrp3*, *Asc*, *Gsdmd*, *Il1b*, and *Il18*) were also induced in the kidneys by LPS and were effectively suppressed by both 10-epi-PDX and RvD5_n-3 DPA_, with RvD5_n-3 DPA_ exerting more pronounced inhibitory effects (Figure 5B). NLRP3 protein expression was also reduced by both oxylipins (Figure S2). Collectively, these data demonstrate that 10-epi-PDX and RvD5_n-3 DPA_ confer robust protection against LPS-induced renal injury and inflammation.

### 2.5. Modulation of Renal Extracellular Matrix Responses by 10-epi-PDX and RvD5_n-3 DPA_

To determine whether LPS challenge altered extracellular matrix (ECM) organization in the kidneys, we assessed renal collagen content, a principal structural component of the ECM, using MT and Sirius Red staining. MT staining revealed a marked expansion of collagen-rich areas in the kidneys of LPS-treated mice. In contrast, kidneys from mice treated with either 10-epi-PDX or RvD5_n-3 DPA_ exhibited preserved tissue architecture and reduced collagen deposition (Figure 5C–F). Consistent with these observations, oxylipin treatment significantly constrained LPS-induced ECM expansion in the kidneys.

### 2.6. Effects of 10-epi-PDX and RvD5_n-3 DPA_ on LPS-Induced Liver Injury and Inflammation

**Figure 6:**
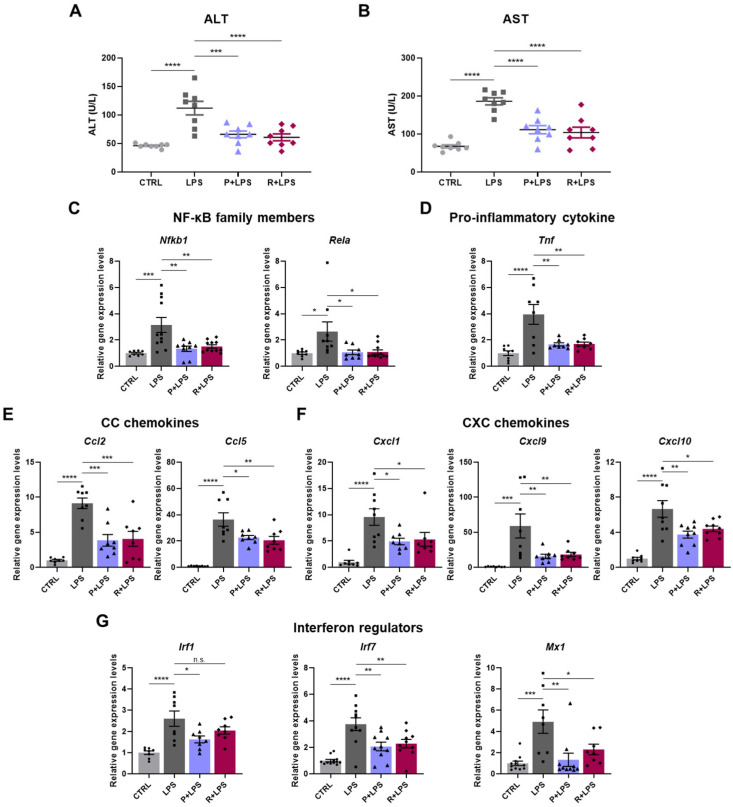
10-epi-PDX and RvD5_n-3 DPA_ inhibit LPS-induced liver injury and inflammation. (**A**,**B**) Serum ALT (**A**) and AST (**B**) levels in mice (CTRL, *n* = 8; LPS, *n* = 8; P+LPS, *n* = 8; R+LPS, *n* = 8). (**C**–**G**) Relative mRNA expression levels of NF-κB subunit genes (**C**), pro-inflammatory cytokines (**D**), CC chemokines (**E**), CXC chemokines (**F**), and interferon-regulated genes (**G**) in liver tissue (CTRL, *n* ≥ 8; LPS, *n* ≥ 8; P+LPS, *n* ≥ 8; R+LPS, *n* ≥ 8). Data are presented as mean ± SEM. Statistical significance was determined by one-way ANOVA followed by Sidak’s post hoc test. * *p* < 0.05, ** *p* < 0.01, *** *p* < 0.001, **** *p* < 0.0001; n.s., not significant.

Because liver injury is a major component of systemic inflammatory responses and contributes to organ dysfunction in sepsis, we next evaluated hepatic responses in LPS-challenged mice [30]. Serum levels of the liver injury markers alanine aminotransferase (ALT) and aspartate aminotransferase (AST) were markedly elevated following LPS administration. Treatment with either 10-epi-PDX or RvD5_n-3 DPA_ significantly reduced these elevations, indicating attenuation of LPS-induced hepatic injury (Figure 6A,B). Histopathological analysis further supported these findings. H&E staining revealed prominent central vein congestion in the livers of LPS-treated mice, which was markedly alleviated by treatment with 10-epi-PDX or RvD5_n-3 DPA_ (Figure S3).

To characterize hepatic inflammatory responses induced by LPS, we examined the expression of inflammation-associated genes in liver tissue. LPS exposure increased the expression of NF-κB-related genes, including *Nfkb1* and *Rela*, along with the pro-inflammatory cytokine *Tnf* (Figure 6C,D). In addition, LPS markedly enhanced hepatic chemokine expression, encompassing both CC chemokines (*Ccl2* and *Ccl5*) and CXC chemokines (*Cxcl1*, *Cxcl9*, and *Cxcl10*) (Figure 6E,F). These immune-related transcriptional responses were substantially downregulated by oxylipin treatment. LPS challenge also induced a pronounced interferon-responsive gene signature in the liver, as evidenced by increased expression of *Irf1*, *Irf7*, and *Mx1* (Figure 6G). This interferon-associated response was effectively attenuated by treatment with 10-epi-PDX or RvD5_n-3 DPA_. Collectively, these results demonstrate that 10-epi-PDX and RvD5_n-3 DPA_ mitigate LPS-induced hepatic injury and inflammatory responses.

### 2.7. Amelioration of Hepatic Inflammation Associated with Reduced NLRP3 Inflammasome Activation by 10-epi-PDX and RvD5_n-3 DPA_

**Figure 7:**
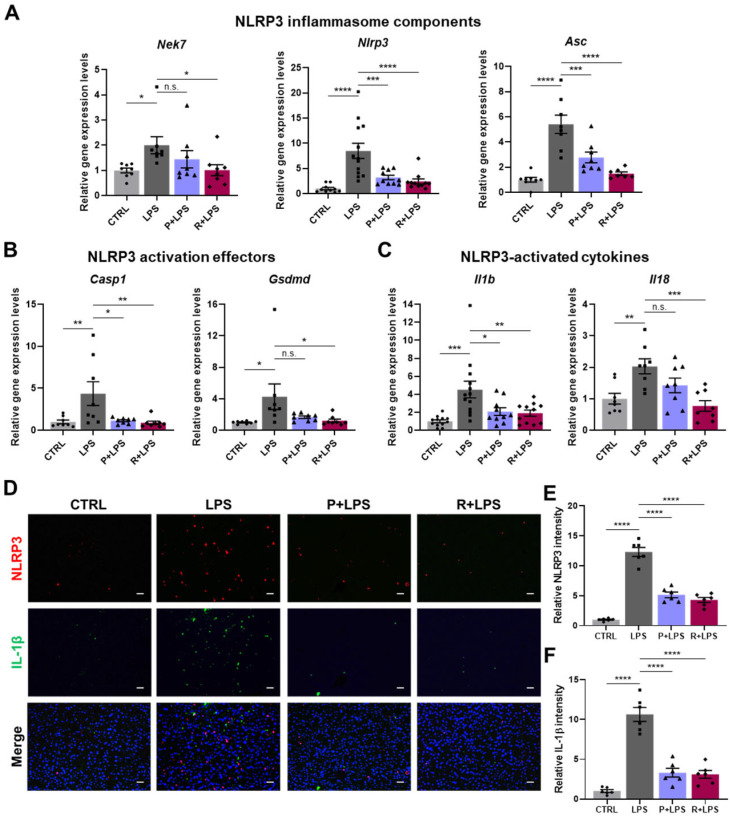
10-epi-PDX and RvD5_n-3 DPA_ modulate NLRP3 inflammasome activation in mouse liver tissue. (**A**–**C**) Relative mRNA expression levels of NLRP3 inflammasome component genes (**A**), NLRP3 activation effector genes (**B**), and NLRP3-activated cytokines (**C**) in liver tissue (CTRL, *n* ≥ 8; LPS, *n* ≥ 8; P+LPS, *n* ≥ 8; R+LPS, *n* ≥ 7). (**D**) Representative immunofluorescence images showing protein expression of NLRP3 and IL-1β in liver tissue (magnification, 200×; scale bar, 100 μm). (**E**,**F**) Quantification of relative NLRP3 (**E**) and IL-1β (**F**) fluorescence intensity (CTRL, *n* = 6; LPS, *n* = 6; P+LPS, *n* = 6; R+LPS, *n* = 6). Data are presented as mean ± SEM. Statistical significance was determined by one-way ANOVA followed by Sidak’s post hoc test. * *p* < 0.05, ** *p* < 0.01, *** *p* < 0.001, **** *p* < 0.0001; n.s., not significant.

In the liver, LPS challenge induced activation of the NLRP3 inflammasome pathway, as reflected by increased expression of genes associated with inflammasome signaling, including *Nek7*, *Nlrp3*, *Asc*, *Casp1*, *Gsdmd*, *Il1b*, and *Il18* (Figure 7A–C). This coordinated induction is consistent with inflammasome priming and activation in hepatic tissue. Notably, treatment with oxylipins substantially attenuated this response, with RvD5_n-3 DPA_ exhibiting the strongest suppressive effect on NLRP3 inflammasome-associated gene expression.

At the protein level, immunofluorescence staining demonstrated marked accumulation of NLRP3 and IL-1β in the livers of LPS-treated mice, consistent with enhanced inflammasome signaling. Administration of either 10-epi-PDX or RvD5_n-3 DPA_ markedly reduced hepatic NLRP3 and IL-1β immunoreactivity, confirming suppression of inflammasome activation at both the transcriptional and protein levels (Figure 7D–F). Collectively, these findings demonstrate that 10-epi-PDX and RvD5_n-3 DPA_ attenuate LPS-induced hepatic inflammasome activation and inflammatory signaling.

## 3. Discussion

Sepsis is a systemic inflammatory syndrome characterized by dysregulated immune activation and progressive multi-organ dysfunction, for which effective disease-modifying therapies remain unavailable [2,4]. However, most studies investigating the anti-inflammatory actions of oxylipins have focused on single-organ models [12,31,32,33,34,35]. Accordingly, rather than restricting our analysis to a single tissue, we evaluated the effects of 10-epi-PDX and RvD5_n-3 DPA_ across the lungs, kidneys, and liver to better reflect multi-organ inflammatory responses relevant to sepsis.

In this study, we demonstrate that the oxylipins 10-epi-PDX and RvD5_n-3 DPA_ attenuate LPS-induced systemic inflammatory pathology in mice, reducing mortality, systemic tissue injury, and inflammatory damage across multiple organs. These protective effects were accompanied by coordinated suppression of inflammatory gene programs and improved histopathological outcomes, supporting a role for oxylipins in limiting inflammation during endotoxemia. Although both mediators conferred broad protection, their effects were not uniform across all inflammatory pathways. RvD5_n-3 DPA_ was associated with a greater reduction in NLRP3 inflammasome-related gene expression, whereas 10-epi-PDX more strongly suppressed interferon-responsive gene programs. These differences likely reflect variations in the magnitude of pathway modulation rather than distinct or mutually exclusive mechanisms of action.

Systemic inflammation associated with sepsis is also frequently linked to brain dysfunction, including neuroinflammation [36]. Systemic LPS administration has been shown to induce inflammatory and immune-related changes in the brain in experimental models [37,38]. Consistent with these observations, LPS administration in our model induced the expression of immune-related genes in the brain, including *Nfkb2*, *Ccl2*, *Ccl5*, *Irf7*, and *Ifih1* (Figure S4), as well as increased expression of *Gfap*, a well-established marker of astrocyte activation during neuroinflammation and brain injury (Figure S5). Under the conditions examined, treatment with 10-epi-PDX or RvD5_n-3 DPA_ did not significantly attenuate these LPS-induced transcriptional changes. These findings may reflect limited central exposure following intraperitoneal administration or the need for alternative dosing strategies.

Notably, additional experiments in which oxylipins were administered after LPS challenge also demonstrated protective effects, supporting their therapeutic potential beyond prophylactic administration (Figure S6). From a therapeutic perspective, our findings indicate that modulation of endogenous lipid-mediator pathways may represent a potential strategy for limiting systemic inflammation and tissue injury in peripheral organs, including the lungs, kidneys, and liver.

However, while the LPS-induced endotoxemia model recapitulates key features of systemic inflammation and multi-organ injury, it does not fully reflect infection-driven sepsis. The cecal ligation and puncture (CLP) model remains the standard for polymicrobial sepsis [39]. Although a certain level of mortality is generally required to reflect the lethality of clinical sepsis, the model used in this study exhibited relatively low lethality. In addition, survival was assessed at 24 h following LPS administration to align with the tissue analysis time point. These factors may limit the interpretation of survival outcomes in terms of therapeutic efficacy. Extending the observation period beyond 24 h could provide additional insight into overall survival outcomes. Nevertheless, the LPS dose and experimental time point were selected to induce robust systemic inflammation and multi-organ injury while maintaining sufficient survival to enable comprehensive analysis of tissue pathology and molecular responses. To further define the translational potential of this approach, future studies should evaluate oxylipin efficacy in polymicrobial infection models that more closely recapitulate the clinical heterogeneity and complexity of human sepsis.

In conclusion, this study demonstrates that 10-epi-PDX and RvD5_n-3 DPA_ mitigate inflammatory injury in key peripheral organs in experimental LPS-induced endotoxemia. By modulating coordinated inflammatory gene programs and limiting tissue damage across organs, these oxylipins provide a foundation for exploring lipid-mediator-based strategies in the prevention and treatment of systemic inflammatory conditions and sepsis-related pathology.

## 4. Materials and Methods

### 4.1. Preparation of 10-epi-PDX and RvD5_n-3 DPA_

10-epi-PDX was synthesized via whole-cell biotransformation using *Escherichia coli* expressing 15S-lipoxygenase from *Archangium violaceum* and purified by preparative HPLC, as described previously [40]. RvD5_n-3 DPA_ was generated by enzymatic conversion of n-3 docosapentaenoic acid (n-3 DPA) using 15S-lipoxygenase, followed by extraction and purification by preparative HPLC, as described previously [13]. The identity of both lipid mediators was confirmed by UV absorbance at 202 nm, and their purity exceeded 90%.

### 4.2. Mice

Seven-week-old male C57BL/6 mice (body weight, 20–25 g) were purchased from Orient Bio (Seongnam, Republic of Korea). Mice were housed in groups of up to five per cage in a specific pathogen-free facility under controlled conditions (12 h light/dark cycle, 22 ± 2 °C, and 50 ± 10% relative humidity), with ad libitum access to standard chow and water. Mice were acclimatized to the housing conditions for one week prior to the initiation of experiments. Mice were randomly assigned to experimental groups. Cage location and order of treatment administration were balanced across groups to minimize potential confounding effects. The investigators responsible for histopathological scoring were blinded to group allocation. The other experimental procedures were performed with knowledge of group allocation. All animal experiments were conducted at the animal care facility of Konkuk University (Seoul, Republic of Korea) in accordance with protocols approved by the Institutional Animal Care and Use Committee (IACUC) (approval no. KU23171; approval date, 17 August 2023).

### 4.3. Injection of 10-epi-PDX, RvD5_n-3 DPA_, and LPS in Mice

Eight-week-old male C57BL/6 mice were randomly assigned to four groups: Vehicle-treated control (CTRL), LPS-treated (LPS), 10-epi-PDX plus LPS-treated (P+LPS), and RvD5_n-3 DPA_ plus LPS-treated (R+LPS). Mice in the P+LPS group received an intraperitoneal injection of 10-epi-PDX (1 mg/kg body weight), while mice in the R+LPS group received RvD5_n-3 DPA_ (1 mg/kg body weight). The oxylipins were initially dissolved in 99.9% ethanol and subsequently diluted in sterile phosphate-buffered saline (PBS) prior to administration, with a final ethanol concentration below 1%. The control and LPS groups received an equivalent volume of vehicle (ethanol diluted in PBS) in place of oxylipins. The dose was selected based on previous studies using oxylipin-related compounds at comparable dose ranges, along with preliminary experiments in our laboratory demonstrating measurable biological effects at this concentration [17]. One hour later, LPS (10 mg/kg) was administered intraperitoneally to the LPS, P+LPS, and R+LPS groups, whereas the CTRL group received PBS. The LPS used in this study was derived from *Escherichia coli* O111:B4 (Sigma-Aldrich, St. Louis, MO, USA). Twenty-four hours after LPS injection, mice were euthanized for sample collection. Serum and tissues, including lung, kidney, liver, and brain, were harvested. Following LPS administration, mice were monitored every 6 h for clinical signs of distress. Humane endpoints were defined as the presence of severe respiratory distress or loss of mobility. All procedures were conducted in accordance with institutional guidelines to minimize animal suffering. The experimental unit was an individual mouse.

### 4.4. Biochemical Analysis

Mouse serum was obtained by centrifugation of whole blood at 2000× *g* for 20 min, and the supernatant was collected. Serum levels of LDH, BUN, creatinine, ALT, and AST were measured using an automated dry chemistry analyzer (FUJI DRI-CHEM 7000i; Fujifilm, Tokyo, Japan).

### 4.5. Histological Analysis

Tissues (lung, kidney, and liver) were fixed in 10% formalin, dehydrated, and embedded in paraffin. Paraffin-embedded tissues were sectioned at 4 µm and stained with H&E, PAS, and MT for lung and kidney, and with H&E for liver. All histological staining was performed by Labcore (Seoul, Republic of Korea).

### 4.6. Histopathological Injury Scoring

Lung injury was evaluated in H&E-stained sections using a scoring system adapted from previous reports [41,42,43]. The assessment criteria included alveolar wall thickening and peribronchial inflammatory infiltration. Each parameter was graded on a scale of 0–4 (0 = none; 1 = mild; 2 = moderate; 3 = severe; 4 = very severe).

Kidney tubular and glomerular injuries were assessed in H&E and PAS-stained sections using scoring systems adapted from previous studies [44,45]. Tubular injury was evaluated based on tubular dilation, tubular atrophy, tubular cast formation, sloughing of tubular epithelial cells, and loss of the brush border or thickening of the tubular basement membrane. Tubular injury was graded on a 0–4 scale according to the percentage of the affected area (0 = normal; 1 = 1–25%; 2 = 26–50%; 3 = 51–75%; 4 = 76–100%).

Glomerular injury was assessed based on mesangial expansion and glomerular sclerosis and graded on a 0–4 scale according to the percentage of the affected area using the same criteria (0 = normal; 1 = 1–25%; 2 = 26–50%; 3 = 51–75%; 4 = 76–100%). The final glomerular damage score was calculated as follows: Final glomerular damage score = 0 × (% grade 0 glomeruli) + 1 × (% grade 1 glomeruli) + 2 × (% grade 2 glomeruli) + 3 × (% grade 3 glomeruli) + 4 × (% grade 4 glomeruli).

Three randomly selected fields per lung and kidney section were evaluated from six mice per group. All histopathological scoring was performed independently by three investigators blinded to the experimental groups.

### 4.7. RNA Isolation

Total RNA was isolated from mouse lung, kidney, liver, and brain tissues using the PureLink RNA Mini Kit (Invitrogen, Carlsbad, CA, USA) following the manufacturer’s instructions. RNA concentration and purity were assessed using a NanoPhotometer N60/N50 spectrophotometer (Implen, München, Germany).

### 4.8. Quantitative Reverse Transcription–Polymerase Chain Reaction (RT-qPCR)

Complementary DNA (cDNA) was synthesized from total RNA using the SuperScript III First-Strand Synthesis Supermix (Invitrogen, Carlsbad, CA, USA). Quantitative PCR was performed using the Power SYBR™ Green PCR Master Mix (Thermo Fisher Scientific, Waltham, MA, USA) on a LightCycler 96 system (Roche, Mannheim, Germany). The gene-specific primers used for qPCR are listed in Table S1. Gene expression levels were normalized to glyceraldehyde-3-phosphate dehydrogenase (*Gapdh*).

### 4.9. Immunofluorescence Assay

Formalin-fixed, paraffin-embedded mouse lung and liver sections (4 µm) were deparaffinized through graded xylene and ethanol washes. Antigen retrieval was performed using a TintoRetriever Heat Retrieval System (Bio SB, Goleta, CA, USA) with 10 mM sodium citrate buffer (pH 6.0) at 95 °C for 10 min. Sections were blocked at 25 °C for 1 h and incubated overnight at 4 °C with primary antibodies against NLRP3 and IL-1β. After washing with PBS, sections were incubated with Alexa Fluor 594-conjugated anti-rabbit IgG (Invitrogen, Carlsbad, CA, USA; #R37117) and Alexa Fluor 488-conjugated anti-mouse IgG (Invitrogen, Carlsbad, CA, USA; #R37120) for 1 h at 25 °C. Samples were mounted using ProLong Gold Antifade Mountant containing DAPI (Invitrogen). Fluorescence images were acquired using a fluorescence microscope (Nikon Eclipse Ts2R; Nikon, Tokyo, Japan), and fluorescence intensity was quantified using ImageJ 1.54s (NIH, Bethesda, MD, USA). Three representative fields per sample were analyzed. A complete list of antibodies is provided in Table S2.

### 4.10. Statistical Analyses

All data are presented as the mean ± standard error of the mean (SEM) from independent biological replicates. Data distribution was assessed using the Shapiro–Wilk test. Statistical analyses were performed using one-way analysis of variance (ANOVA) followed by Sidak’s post hoc test. All statistical analyses were conducted using GraphPad Prism 10.0 (GraphPad Software, San Diego, CA, USA). Outliers were identified using Grubbs’ test and excluded prior to statistical analysis. No animals were excluded for other reasons, and no additional inclusion or exclusion criteria were applied. Detailed information on specific group comparisons is provided in the figure legends. The final sample size (*n*) for each experimental group is listed in Table S3. Sample sizes were determined to obtain reliable and reproducible results for biochemical, histological, and gene expression analyses.

### 4.11. Outcome Measures

The primary outcome measure was the extent of organ injury following LPS challenge, assessed by histopathological scoring and serum biochemical markers. Secondary outcomes included survival, body weight changes, and inflammatory gene and protein expression.

## Data Availability

The original contributions presented in this study are included in the article/Supplementary Materials. Further inquiries can be directed to the corresponding author.

## Supplementary Materials

The following supporting information can be downloaded at: https://www.mdpi.com/article/10.3390/ijms27083356/s1.

## Abbreviations

The following abbreviations are used in this manuscript:

10-epi-PDX	10-epi-Protectin DX
RvD5_n-3 DPA_	Resolvin D5_n-3 DPA_
LPS	Lipopolysaccharide
PDX	Protectin DX
CTRL	Control
LDH	Lactate dehydrogenase
ALI	Acute lung injury
ARDS	Acute respiratory distress syndrome
H&E	Hematoxylin and eosin
PAS	Periodic acid–Schiff
MT	Masson’s trichrome
AKI	Acute kidney injury
BUN	Blood urea nitrogen
ECM	Extracellular matrix
ALT	Alanine aminotransferase
AST	Aspartate aminotransferase
RT-qPCR	Quantitative reverse transcription–polymerase chain reaction
