# Supplementary material for: 10-epi-Protectin DX and Resolvin D5n-3 DPA Attenuate Multi-Organ Inflammatory Injury in an LPS-Induced Murine Endotoxemia Model
Source: Int J Mol Sci. 2026 Apr 8;27(8):3356. doi: 10.3390/ijms27083356 (PMC13116450; doi:10.3390/ijms27083356)
Supplement: Supplementary file 1 [file ijms-27-03356-s001.zip › ijms-4196627-supplementary.pdf]

Supplementary Materials

10-epi-Protectin DX and Resolvin D5$_{n-3\ DPA}$ attenuate multi-organ inflammatory injury in an LPS-induced murine endotoxemia model

Suyeon Kim *et al.*

Supplementary Methods

Western blotting

Mouse kidney tissues were prepared using RIPA buffer supplemented with protease inhibitors (Roche, Basel, Switzerland). Equal amounts of protein were separated on 8% SDS-PAGE gels with AccuLadder™ 3-color Prestained Protein size marker (Bioneer, Daejeon, Republic of Korea), transferred onto nitrocellulose membranes and detected using enhanced chemiluminescence (Thermo Fisher Scientific, Waltham, MA, USA) and imaged using the ChemiDoc imaging system (Bio-Rad, Hercules, CA, USA). An anti-NLRP3 antibody was used (Novus Biologicals, Centennial, CO, USA; #NBP2-12446). β-actin was used as a loading control.

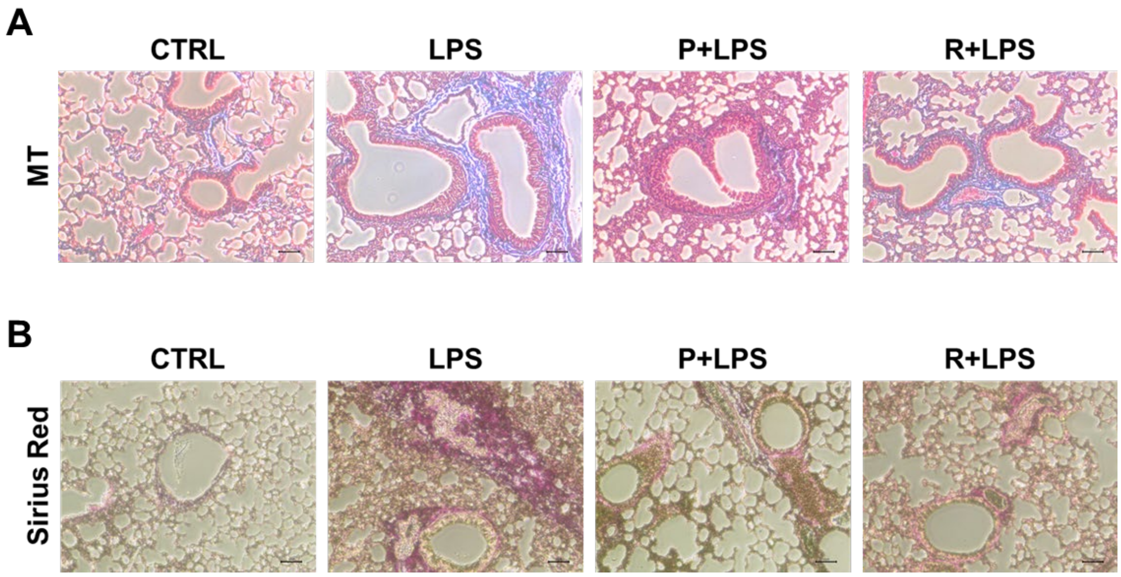

Figure S1. Histological analyses of mouse lung tissue.

(**A, B**) Representative lung sections from the CTRL, LPS, P+LPS, and R+LPS groups stained with Masson's trichrome (MT) (**A**) and Sirius Red (**B**). Images were acquired at 100× magnification (scale bar = 10 μm).

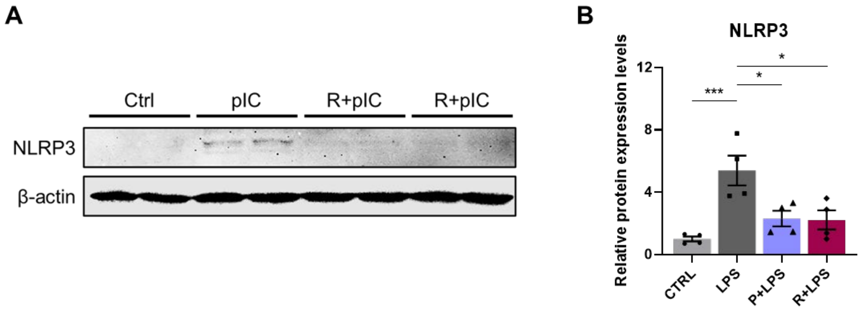

Figure S2. 10-epi-PDX and RvD5$_{n\text{-}3\ DPA}$ mitigate LPS-induced NLRP3 protein expression in mouse kidney tissue.

(**A**) Western blot analysis of NLRP3. (**B**) Quantification of relative NLRP3 protein levels normalized to β-actin (CTRL, n = 4; LPS, n = 4; P+LPS, n = 4; R+LPS, n = 4). Data are presented as mean ± SEM. Statistical significance was determined by one-way ANOVA followed by Sidak's post hoc test. * $p < 0.05$, *** $p < 0.001$.

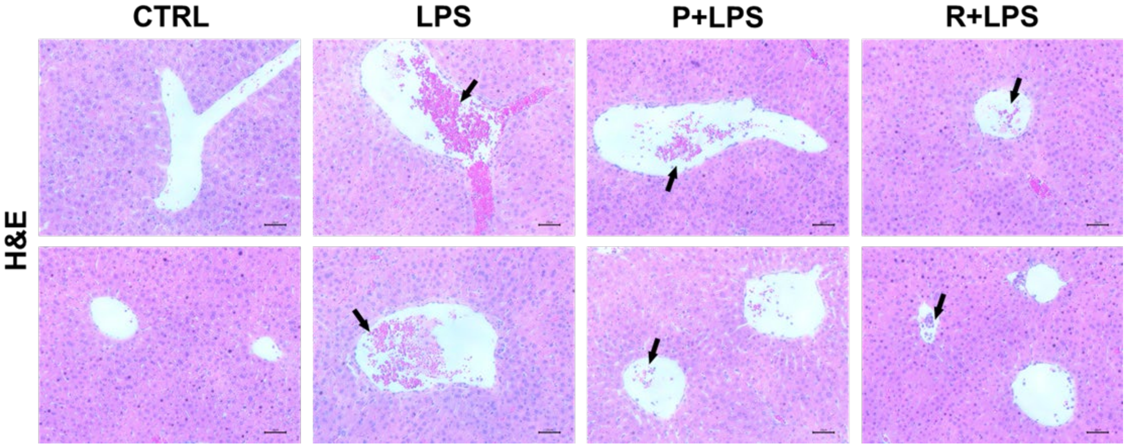

Figure S3. 10-epi-PDX and RvD5$_{n\text{-}3\ DPA}$ attenuate LPS-induced hepatic central vein congestion.

Representative H&E-stained liver sections from the CTRL, LPS, P+LPS, and R+LPS groups. Images were acquired at 100× magnification (scale bar = 10 μm). Central vein congestion with erythrocyte accumulation is indicated by black arrows.

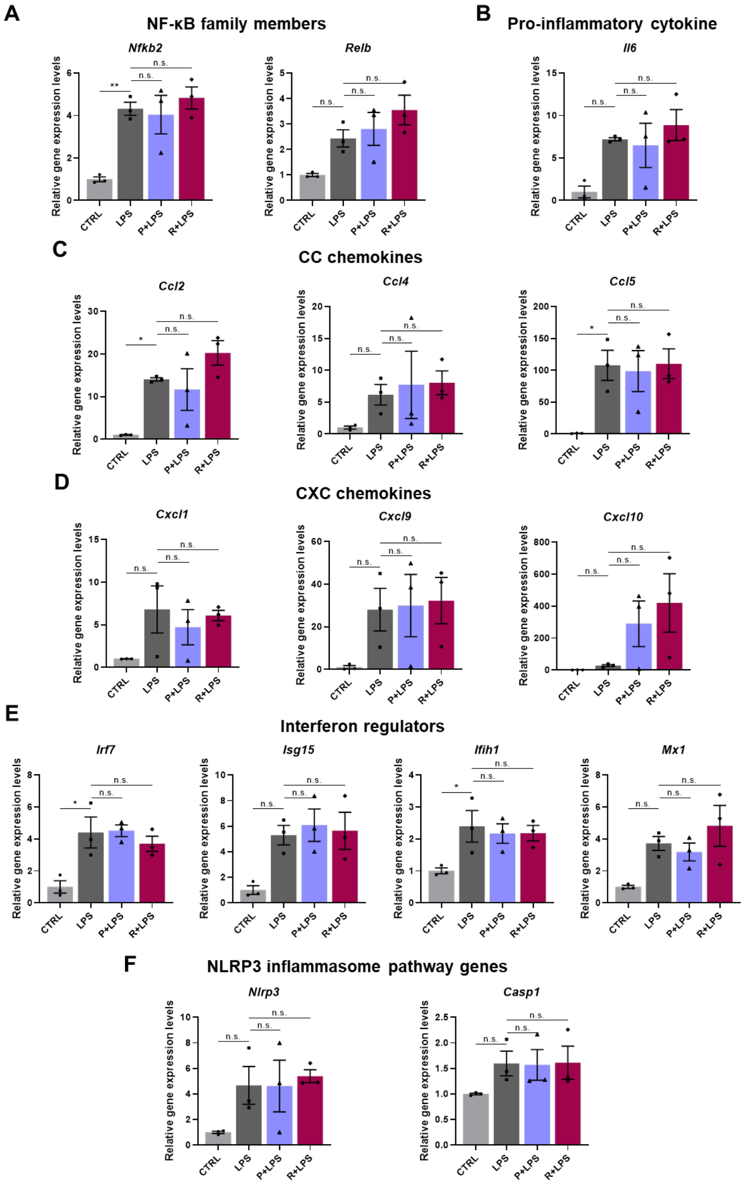

Figure S4. Effect of 10-epi-PDX and $RvD5_{n\text{-}3\ DPA}$ on LPS-induced neuroinflammatory gene expression in brain tissue.

(**A**–**F**) Relative mRNA expression levels of NF-κB subunit genes (**A**), pro-inflammatory cytokines (**B**), CC chemokines (**C**), CXC chemokines (**D**), interferon-regulated genes (**E**), and NLRP3 inflammasome-related genes (**F**) in brain tissue. Gene expression was normalized to *Gapdh* (CTRL, n = 3; LPS, n = 3; P+LPS, n = 3; R+LPS, n = 3). Data are presented as mean ± SEM. Statistical significance was determined by one-way ANOVA followed by Sidak's post hoc test. * $p < 0.05$, n.s., not significant.

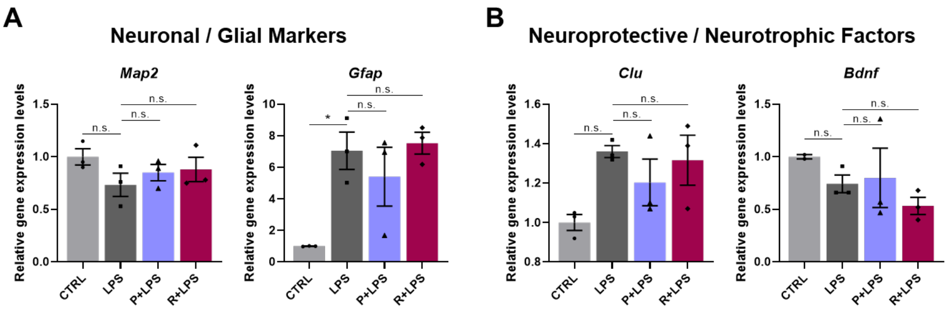

Figure S5. Effect of 10-epi-PDX and RvD5$_{n\text{-}3\ DPA}$ on LPS-induced neural stress-related gene expression in brain tissue.

(**A**, **B**) Relative mRNA expression levels of neuronal- and glial-related genes (**A**) and neuroprotective- and neurotrophic-related genes (**B**) in brain tissue. Gene expression was normalized to *Gapdh* (CTRL, n = 3; LPS, n = 3; P+LPS, n = 3; R+LPS, n = 3). Data are presented as mean ± SEM. Statistical significance was determined by one-way ANOVA followed by Sidak's post hoc test. * $p < 0.05$, n.s., not significant.

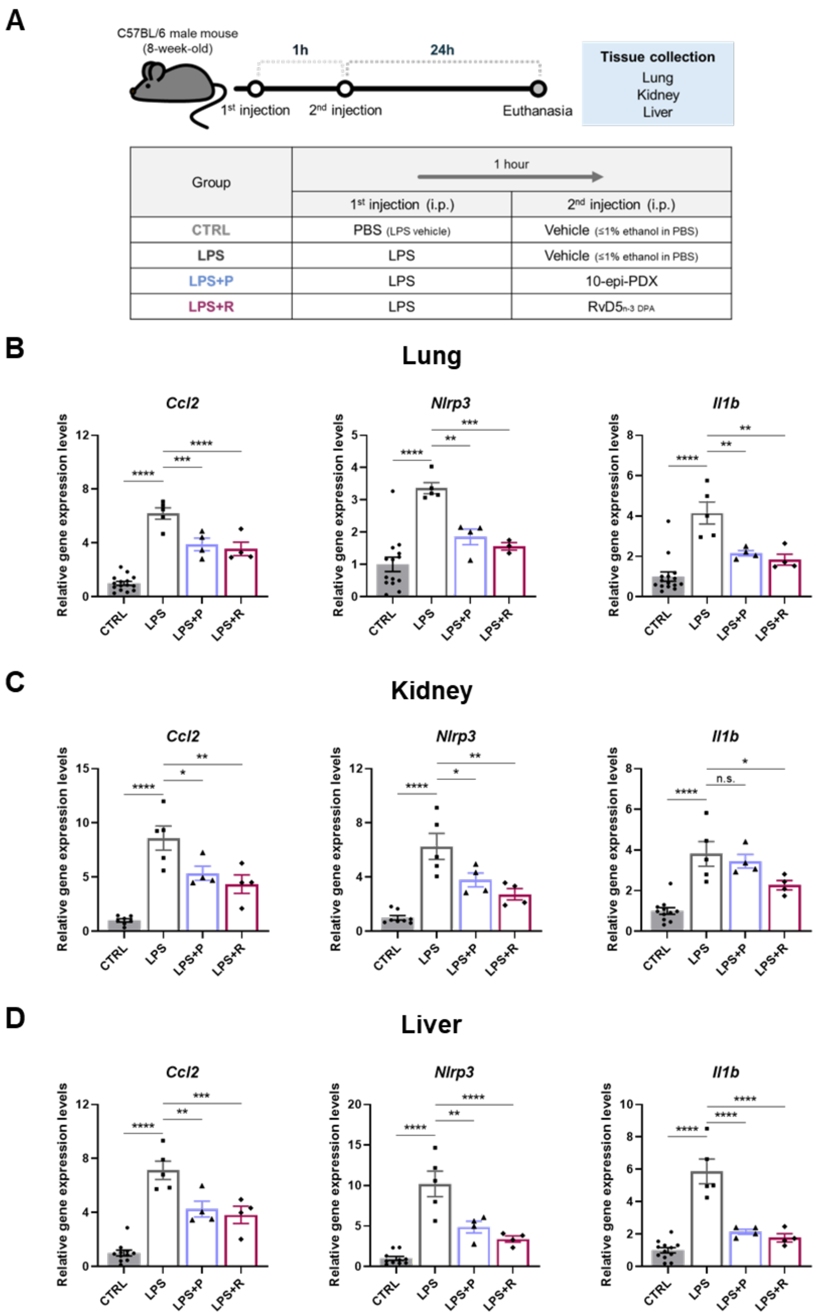

Figure S6. Post-treatment with 10-epi-PDX and RvD5$_{\text{n-3 DPA}}$ alleviates LPS-induced inflammatory responses and multi-organ injury in mice.

(**A**) Schematic of the experimental design used to evaluate the therapeutic effects of 10-epi-PDX and RvD5$_{\text{n-3 DPA}}$ in an LPS-induced endotoxemia model. (**B–D**) Relative mRNA expression levels of *Ccl2*, *Nlrp3*, and *Il1b* in mouse lung (**B**), kidney (**C**), and liver (**D**) tissues (CTRL, $n \geq 8$; LPS, $n = 5$; P+LPS, $n = 4$; R+LPS, $n = 4$). Data are presented as mean ± SEM. Statistical significance was determined by one-way ANOVA followed by Sidak's post hoc test. * $p < 0.05$, ** $p < 0.01$, *** $p < 0.001$, **** $p < 0.0001$, n.s., not significant.

Table S1. Gene-specific primer sequences used for RT-qPCR analysis

Target gene		Primer sequences
Gapdh	Forward	5'-TGTGTCCGTCGTGGATCTGA-3'
	Reverse	5'-TTGCTGTTGAAGTCGCAGGAG-3'
Nfkb1	Forward	5'-GAAATTCCTGATCCAGACAAAAAC-3'
	Reverse	5'-ATCACTTCAATGGCCTCTGTGTAG-3'
Nfkb2	Forward	5'-CTGGTGGACACATACAGGAAGAC-3'
	Reverse	5'-ATAGGCACTGTCTTCTTTCACCTC-3'
Rela	Forward	5'-CTTCCTCAGCCATGGTACCTCT-3'
	Reverse	5'-CAAGTCTTCATCAGCATCAAACTG-3'
Relb	Forward	5'-CTTTGCCTATGATCCTTCTGC-3'
	Reverse	5'-GAGTCCAGTGATAGGGGCTCT-3'
Tnf	Forward	5'-AGCCCCCAGTGTGTATCCTT-3'
	Reverse	5'-ACAGTCCAGGTCACTGTCCC-3'
Il6	Forward	5'-AGTTGCCTTCTTGGGACTGA-3'
	Reverse	5'-TCCACGATTTCCCAGAGAAC-3'
Ccl2	Forward	5'-GCTACAAGAGGATCACCAGCAG-3'
	Reverse	5'-GTCTGGACCCATTCCTTCTTGG-3'
Ccl4	Forward	5'-ACCCTCCCACTTCCTGCTGTTT-3'
	Reverse	5'-CTGTCTGCCTCTTTTGGTCAGG-3'
Ccl5	Forward	5'-CCTGCTGCTTTGCCTACCTCTC-3'
	Reverse	5'-ACACACTTGGCGGTTCCTTCGA-3'
Cxcl1	Forward	5'-TCCAGAGCTTGAAGGTGTTGCC-3'
	Reverse	5'-AACCAAGGGAGCTTCAGGGTCA-3'
Cxcl9	Forward	5'-CCTAGTGATAAGGAATGCACGATG-3'
	Reverse	5'-CTAGGCAGGTTTGATCTCCGTTC-3'
Cxcl10	Forward	5'-CCAAGTGCTGCCGTCATTTTC-3'
	Reverse	5'-GGCTCGCAGGGATGATTTCAA-3'
Irf1	Forward	5'-TCCAAGTCCAGCCGAGACACTA-3'
	Reverse	5'-ACTGCTGTGGTCATCAGGTAGG-3'
Irf7	Forward	5'-CCTCTGCTTTCTAGTGATGCCG-3'
	Reverse	5'-CGTAAACACGGTCTTGCTCCTG-3'
Ifit1	Forward	5'-TACAGGCTGGAGTGTGCTGAGA-3'
	Reverse	5'-CTCCACTTTCAGAGCCTTCGCA-3'
Ifit2	Forward	5'-CGAACTACCGTCTGGATGACTG-3'
	Reverse	5'-CTTCAACCAGCGCCATTGCTTG-3'
Ifit3	Forward	5'-GCTCAGGCTTACGTTGACAAGG-3'
	Reverse	5'-CTTTAGGCGTGTCCATCCTTCC-3'
Mx1	Forward	5'-TGGACATTGCTACCACAGAGGC-3'

	Reverse	5'-TTGCCTTCAGCACCTCTGTCCA-3'
Isg15	Forward	5'-CATCCTGGTGAGGAACGAAAGG-3'
	Reverse	5'-CTCAGCCAGAACTGGTCTTCGT-3'
Rsad2	Forward	5'-GGAAGGTTTTCCAGTGCCTCCT-3'
	Reverse	5'-ACAGGACACCTCTTTGTGACGC-3'
Nek7	Forward	5'-CTGGTCTCTTGGCTGTCTGCTA-3'
	Reverse	5'-CCTCCGAATAGTGATCTGACGG-3'
Nlrp3	Forward	5'-TCACAACGCCCAGGAGGAA-3'
	Reverse	5'-AAGAGACCACGGCAGAAGCTAG-3'
Asc	Forward	5'-CTGCTCAGAGTACAGCCAGAAC-3'
	Reverse	5'-CTGTCCTTCAGTCAGCACACTG-3'
Casp1	Forward	5'-GGCACATTTCCAGGACTGACTG-3'
	Reverse	5'-GCAAGACGTGTACGAGTGGTTG-3'
Gsdmd	Forward	5'-GGTGCTTGACTCTGGAGAACTG-3'
	Reverse	5'-GCTGCTTTGACAGCACCGTTGT-3'
Il1b	Forward	5'-CTTCAGGCAGGCAGTATCACTC-3'
	Reverse	5'-TTGTTGTTCATCTCGGAGCC-3'
Il18	Forward	5'-GACAGCCTGTGTTCGAGGATATG-3'
	Reverse	5'-TGTTCTTACAGGAGAGGGTAGAC-3'
Ifih1	Forward	5'-TGCGGAAGTTGGAGTCAAAGCG-3'
	Reverse	5'-CACCGTCGTAGCGATAAGCAGA-3'
Map2	Forward	5'-GCTGTAGCAGTCCTGAAAGGTG-3'
	Reverse	5'-CTTCCTCCACTGTGGCTGTTTG-3'
Gfap	Forward	5'-CACCTACAGGAAATTGCTGGAGG-3'
	Reverse	5'-CCACGATGTTCCTCTTGAGGTG-3'
Clu	Forward	5'-GATGATCCACCAGGCTCAACAG-3'
	Reverse	5'-ACACAGTGCGGTCATCTTCACC-3'
Bdnf	Forward	5'-GGCTGACACTTTTGAGCACGTC-3'
	Reverse	5'-CTCCAAAGGCACTTGACTGCTG-3'

Table S2. Primary antibodies used for immunofluorescence staining

Antibody	Host	Manufacturer	Catalog number
Anti-NLRP3/NALP3	Rabbit	Novus Biologicals	#NBP2-12446
Anti-IL-1 beta/IL-1β	Mouse	Santa Cruz	#sc-52012

Table S3. Sample sizes (*n*) for each figure panel

Figure	Panel	Graph	*n*			
			CTRL	LPS	P+LPS	R+LPS
Fig. 1	B		21	26	23	23
	C		21	26	23	23
	D		21	24	23	23
	E		8	8	8	8
Fig. 2	B		6	6	6	6
	C	*Nfkb2*	15	14	8	10
		Relb	15	12	11	12
	D	*Tnf*	10	10	9	9
		Il6	10	10	9	9
	E	*Ccl2*	15	13	11	9
	F	*Cxcl1*	11	12	8	8
		Cxcl9	10	12	10	11
		Cxcl10	8	9	9	9
	G	*Irf7*	12	10	9	9
		Ifit1	8	8	8	8
		Ifit2	8	8	8	8
		Isg15	11	9	8	8
		Rsad2	8	8	8	8
Fig. 3	A	*Nek7*	8	8	8	8
		Nlrp3	14	11	10	12
		Casp1	8	8	8	9
		Il1b	15	14	9	14
		Il18	8	8	8	8
	C		6	6	6	6
	D		6	6	6	6
	F		6	6	6	6
	H		6	6	6	6
	I		6	6	6	6
Fig. 4	A		8	8	8	8
	B		8	8	8	8
	D		6	6	6	6

	F		6	6	6	6
	G	*Nfkb1*	8	8	8	8
		Nfkb2	8	8	8	8
	H	*Tnf*	8	8	8	8
		Il6	8	8	8	8
	I	*Ccl2*	8	8	8	8
		Ccl5	8	8	8	8
	J	*Cxcl1*	8	9	8	8
		Cxcl9	8	8	8	8
		Cxcl10	8	9	9	9
Fig. 5	A	*Ifit1*	8	8	8	8
		Ifit2	8	8	8	8
		Ifit3	8	8	8	8
		Mx1	8	8	8	8
		Rsad2	8	8	8	8
	B	*Nlrp3*	8	8	8	8
		Asc	8	8	8	8
		Gsdmd	8	9	8	9
		Il1b	11	10	8	12
		Il18	8	8	8	8
	D		6	6	6	6
	F		6	6	6	6
Fig. 6	A		8	8	8	8
	B		8	8	8	8
	C	*Nfkb1*	8	11	9	11
		Rela	8	9	8	11
	D	*Tnf*	8	8	8	8
	E	*Ccl2*	11	8	8	8
		Ccl5	8	9	8	8
	F	*Cxcl1*	8	8	8	8
		Cxcl9	8	13	8	8
		Cxcl10	14	9	8	8
	G	*Irf1*	8	8	8	8
		Irf7	12	10	10	10
		Mx1	11	8	10	8

Fig. 7	A	*Nek7*	8	8	8	8
		Nlrp3	10	13	10	11
		Asc	8	8	8	7
	B	*Casp1*	8	8	8	8
		Gsdmd	8	8	8	8
	C	*Il1b*	12	13	10	11
		Il18	8	8	8	8
	E	6	6	6	6	6
	F	6	6	6	6	6
